# The relationship between self-consciousness and depression in college students: the chain mediating effect of meaning life and self-efficacy, with the moderating effect of social support

**DOI:** 10.1186/s12889-024-18263-w

**Published:** 2024-03-13

**Authors:** Ye Yuan, Daili Wu, Zhongnong Chen, Daile Chen, Qiang Zhou, Jaesik Jeong, Yanling Tu

**Affiliations:** 1 School of Mental Health, Wenzhou Medical University, Wenzhou, 325035 China; 2 Zhejiang Procince Clinical Research Center for Mental Disorders, The Affiliated Wenzhou Kangning Hospital, Institute of Aging, Key Laboratory of Alzheimer’s Disease of Zhejiang Province, Wenzhou Medical University, Wenzhou, 325035 China; 3 Department of Mathematics and Statistics, Chonnam National University, Gwangju, 61186 South Korea; 4 School of Stomatology, Wenzhou Medical University, Wenzhou, 325035 China; 5 Wenzhou University of Technology, wenzhou, China

**Keywords:** Self-consciousness, Depression, Life meaning, Self-efficacy, Social support

## Abstract

**Background:**

This study aimed to investigate the impact of self-consciousness on depression of college students, and mainly focus on confirming the mediator role of life meaningful and self-efficacy, as well as the moderator role of social support.

**Methods:**

In the present study, convenient sampling method was adopted, 583 college students were recruited from Harbin city and Wenzhou city in China. All students were assessed using self-assessment scales, including self-consciousness scale, life meaningful scale, self-efficacy scale, social support scale, and self-rating depression scale. Descriptive statistical analysis and correlation analysis, structural equation model analysis were conducted by SPSS 25.0 and M-plus.

**Results:**

Results showed that self-consciousness was negatively related to depression, life meaningful and self-efficacy partially mediated the relation between self-consciousness and depression. Moderated mediation analysis further indicated that the relation between self-efficacy and depression were moderated bu social support. Compare with college students who had high social support, depression in those with low social support was more susceptible to the effect of self-efficacy.

**Conclusion:**

These findings imply that college students with low levels of self-consciousness are more easy to be depressive, enhancing their sense of life meaning and self-efficacy can effectively alleviate depression, and college student with high social support can benefit more from self-efficacy. Therefore we should pay more attention to the mental health problems of low levels self-consciousness college students in university.

## Introduction

Depression is a psychological disorder characterized by a negative mood, lack of motivation, and a sense of life meaningless. It is accompanied by the experience of emotions such as anger, sadness, self-guilt and shame in different situations (DMS-5 diagnostic). One of the core symptoms of depressive states of mind that do not meet the diagnostic criteria for depression is negative evaluations of self [1]. Such self-defeating thoughts can further aggravate the patient’s mood and trigger additional symptoms. Therefore, It may has a significant correlation between depression and self-consciousness [2–3].

Self-consciousness is individuals’ understanding of themselves and their relationship with their surroundings, reflecting their position in the environment, society and values. It is a guarantee for individuals to achieve socialization goals and improve their personality traits [4]. Studies showed that there may be a causal relationship between self-consciousness and depression in college students [5]. Because negative self-attributions may impede effective coping with failure or loss, leading to poor regulation of the discrepancy between ideal and real selves, which can trigger depression [6]. It has also been shown that when shame is controlled, the link between guilt and depression disappears or becomes smaller [7]. Enhancing pride can promote positive emotions and thinking about themselves, enhancing social value and mental health [8]. Therefore, this study hypothesizes that positive self-consciousness levels may negatively predict the performance of depression in college students.

The sense of meaning in life refers to individuals’ subjective experiences as valuable, purposeful and directional [9]. Individuals who are faced with life’s challenges take the initiative to create and find meaning in their lives [10]. Numerous studies have shown that sense of meaning in life have positive effects, for example, a sense of life meaningful can adjust health-related attitudes and behaviors, promoting health for students [11]. In terms of mental health, college students with a sense of meaning in life have a healthier psychological state are more satisfied with life, are happier and more optimistic about the future, and have better psychological adaptability [12, 13]. When college students face adversity, they can reduce psychological pain and the adverse effects of traumatic experiences in life by pursuing a sense of life purpose [14]. Conversely, college students who lack of a sense of life meaningful in experience more lonely, and have a higher levels of anxiety and depression [15]. A sense of meaning in life is a direct reflection of self-awareness, and a blurred sense of self not only diminishes an individual’s sense of meaningfulness in connecting with others, but also diminishes the individual’s pursuit of life’s purpose [16]. So in this hypothesis self-consciousness may contribute to depression in college students by impairing their sense of life meaningful.

Self-efficacy refers to subjective judgment and confidence level in one’s ability to complete a task [17]. Many studies suggest that there is a significant correlation between adolescent self-efficacy and depression [18]. When college students face difficulties at this stage of development, such as family environment difficulties, academic difficulties and employment difficulties, self-efficacy can promote their adaptive development [19, 20]. College students with higher self-efficacy always view threat situations as challenges, and then set high goals and maintain strong commitment. They can maintain hope and continue working hard when faced with failure, which will in turn reduce the risk of depression [21]. In contrast, college students with lower self-efficacy often avoid difficulties and experience more negative emotions and tension, at last the risk of depression increased [22]. Therefore, the present study hypothesized that self-consciousness can reduce the risk of depression by enhancing college students’ self-efficacy.

Some researches have shown that there is a correlation between self-efficacy and sense of meaning in life [23]. Self-efficacy is a kind of optimistic self-belief [24], when college students exert their initiative to persist in pursuing a goal until the task is achieved, self-efficacy can be improved, thus a higher sense of meaning in life can be experienced [25]. Studies have explored the relationship between self-efficacy and sense of meaning in specific functional domains, such as social self-efficacy, social self-efficacy, occupational decision-making self-efficacy, and network efficacy [26]. Self-efficacy is a kind of exploratory self-identity of an individual’s own ability, and the degree of this identity is a necessary condition and an important element that affects the individual’s experience of meaning in life [27]. Academic self-efficacy is a manifestation of self-efficacy in the academic domain, it is the subjective evaluation of students’ self-efficacy [28]. The higher the students’ evaluation of their self-efficacy, the more conducive they will be to enhancing their self-worth (e.g., sense of meaning in life, self-esteem, etc.) [29].

Social support is the perception of emotional experiences related to support, respect and understanding from the outside world [30]. Studies showed that the primary effect model holds that social support maintains good emotional experiences, reduces negative emotions and reduces the risk of depression [31]. Social support has a negative predictive effect on depression [32]. College students with lower self-efficacy can effectively alleviate depression by using external support to buffer the impact of stress time on their emotions, enhancing their confidence and endurance when facing challenges and alleviating depression [33]. Therefore, this study hypothesis that social support would moderate the relationship between self-efficacy and depression.

In summary, this study proposes the following research hypotheses:

1. Self-consciousness can negatively predict the risk of depression in college students.
2. The life meaningful and self-efficacy mediate the relationship between self-consciousness and depression.
3. Social support moderates the relationship between self-efficacy and depression.

**Fig. 1:**
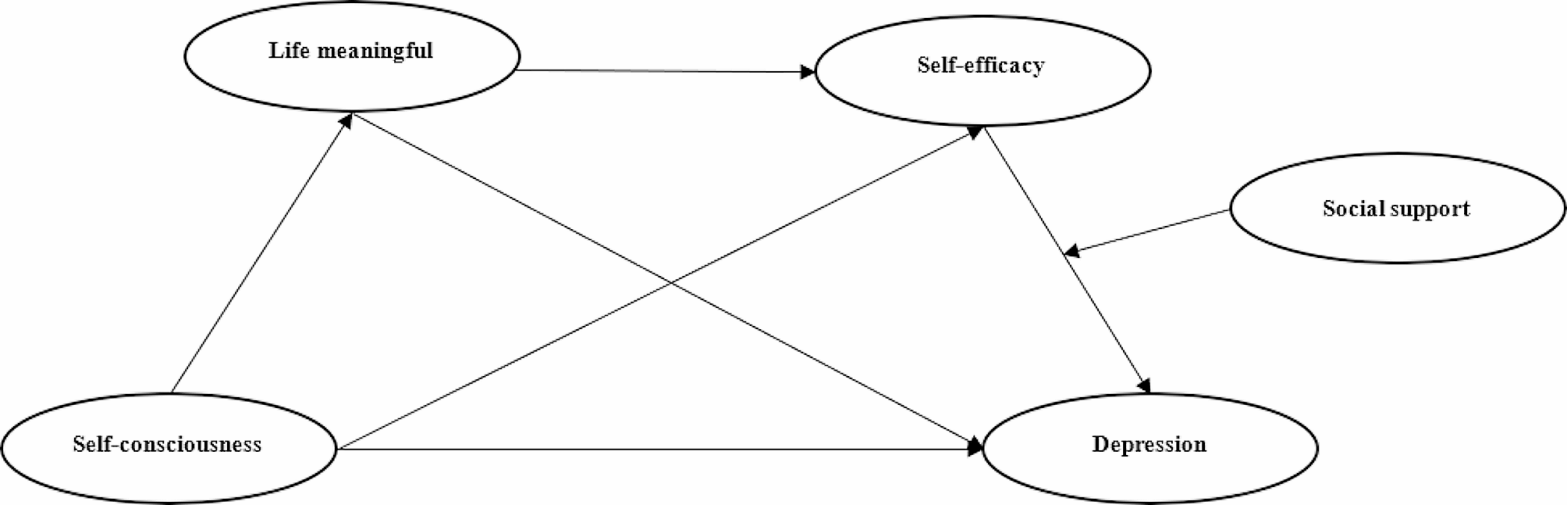
Theoretical model

Based on the above three theoretical assumptions, this study constructs a theoretical hypothesis model, as shown in Fig. 1.

## Methods

### Participants

In accordance with the Helsinki Declaration, the study was conducted and revised under the approval of the ethics committees of Wenzhou Medical University. The questionnaire content was accessed via a QR code through the We-Chat Star questionnaire, researchers administered self-report measurement during regular school hours in the classroom. The purpose of the survey was explained in the questionnaire instructions before conducting the survey, and guarantee the confidentiality of participants’ information. Students used their mobile phones to scan the QR code and to finish the survey. Before participation, everyone either provided their oral consent by raising their hands or checked a box indicating informed consent on the online questionnaires. Participants were given instructions regarding each scale before rating the items. They were invited to fill out the five self-report questionnaires under the principle of voluntary, anonymous and confidential.

Use the convenient sampling method, 600 undergraduate students were recruited. Variables such as gender and grade were controlled. After excluding 17 invalid questionnaires caused by data missing or regular responses, 583 valid questionnaires were collected, yielding a valid response rate of 97.2%.

### Measures

#### Self-consciousness scale

The self-consciousness scale was developed by Fenigstein [34]. It involves personal self-consciousness, public self-consciousness, and social anxiety. The scale has 17 items, with two reverse and 15 positive ratings. The scale adopts a five-point scale, where 1 represents “completely inconsistent”, 2 represents “not very consistent”, 3 represents “unclear”, 4 represents “quite consistent” and 5 represents “very consistent”. High scores indicate clear self-understanding. The internal consistency coefficient of the scale is 0.819. Cronbach’s α was 0.83 in this study.

#### Life meaning scale

Life meaning scale was developed by Steger [35]. The internal consistency coefficients of the total scale, MLQ-P, and MLQ-S 0.868, 0.811, and 0.841, respectively, all greater than 0.8, indicating good consistency. High scores indicate a strong sense of meaning in life. This table consists of ten items and is divided into two sub-scales: (1) the presence of meaning (MLQ-P), which includes five items. (2) The search for meaning (MLQ-S) contains five items. The scale uses a seven-point scoring method, ranging from 1 to 7 points, with 1 point indicating “completely inconsistent” and 7 points indicating “completely consistent.” In the present study, the Cronbach’s α of the scale was 0.89.

#### Social support scale

The social support rating scale was developed by Shuiyuan Xiao [36], with a total of ten items, including three dimensions: objective support (three items), subjective support (four items), and utilization of social support (three items). Items 1–4 and 8–10 of the scale have only one option per item, and the total score is calculated on a four-point scale. The fifth point, items A, B, C, and D, were counted as the total score. The answer “from the following sources” scores a few points if there are several sources. High scores indicate high social support. A total score of less than 22 points indicates a low level, a score of 23–44 points indicates a moderate level and a score of 45–66 points indicates a high level. In the present study, the Cronbach’s α of the scale was 0.94.

#### General self-efficacy scale

Professor Ralf Schwarzer developed the general self-efficacy scale [37]. It consists of ten items that involve confidence when encountering setbacks or difficulties. The scale uses a four-point scoring system, where 1 represents completely incorrect, 2 represents correct, 3 represents mostly correct, and 4 represents completely correct. One-tenth of the total score of the ten questions answered by the subject is the final score, with a score range of 1–4 points. The critical score is 2.5 points. Scores below 2.5 points indicate that the subject’s general self-efficacy is low. The retest confidence of this scale was 0.83, and the half reliability was 0.82. The Cronbach’s α of was 0.88 in this study.

#### Self-rating depression scale

The self-rating depression scale was developed by Zung [38]. It included 20 items (ten positive and ten negative scores), including two for psycho-emotional symptoms, eight for physical disorders, two for Psychomotor disorders, and eight for depressive psychological disorders. Each project consists of four levels of scoring, with 1 representing “no or very little time,” 2 representing “a small portion of time,” 3 representing “a considerable amount of time,” and 4 representing “most or all of the time.” The standard score is the integer part obtained by multiplying the rough score by 1.25. A score of 53–62 indicates mild depression, 63–72 indicates moderate depression, and above 73 indicates severe depression. The Cronbach’s α was 0.91 in this study.

### Statistical analyses

All statistical analyses were performed using SPSS 22.0 for descriptive and correlation analysis of the variables. M-plus 7.0 was used to test the hypothesis on the mediating and moderated effects. The mediating effect of life meaningful and self-efficacy was estimated by the bootstrap procedure (*n* = 5000), which is considered to be the most accurate by Hayes. Three direct effect and the total effect (the direct effect of the independent variable on the mediator, the direct effect of the independent variable on the dependent variable, the direct effect of the mediator on the dependent variable, and the total effect of the independent variable on the dependent variable) were calculated automatically. Full mediation or partial mediation depends on whether the effect of the independent variable on the dependent variable is significant when the mediating effect is significant. To test for a moderating effect, the effect of the independent variable on the dependent variable, the effect of the moderator on the dependent variable, and the interaction effect on the dependent variable all need to be significant. Age, gender, grade, major, family member and registered permanent residence were adjusted in the moderation and mediation models. AMOS software was used to draw the moderating effect map. *P* < 0.05 indicates a statistically significant difference.

The data collection relied on self-reporting, which may lead to CMV issues. To improve the study’s rigor, it is necessary to conduct CMV testing on the data, using Harman’s single factor test method to perform non-rotated principal component factor analysis on all variables. The variance of the primary factor was 17%, which is less than the critical value of 40%. Therefore, the data used in this study are scientifically accurate.

## Results

### The information of the participants

The age distribution of the participants was 18–24 years, with 279 males (47.8%) and 304 females (52.2%). There were 143 freshmen (24.5%), 159 sophomores (27.3%), 180 juniors (30.8%), and 102 seniors (17.4%). There were 118 humanities majors (20.2%), 222 science and engineering majors (38.1%), 101 art and business majors (17.3%), and 142 medical majors (24.4%). Participants information are shown in Table 1.

**Table 1:** Description of the study variable and the distribution of depression students

Subject	Category	Number	Depression (score < 53)	Depression (score ≥ 53)	*P*
Gender	Male	279 (47.86%)	269	10	0.000^**^
	Female	304 (52.14%)	279	25	
Age	18–20	201 (34.48%)	189	12	0.003^**^
	20–22	189 (32.42%)	179	10	
	22–24	193 (33.10%)	180	13	
Year	Freshmen	143 (24.53%)	133	10	0.013^**^
	Sophomores	159 (27.27%)	151	8	
	Juniors	180 (30.87%)	172	7	
	Seniors	102 (17.50%)	92	10	
Major	Natural Sciences	98 (16.81%)	92	6	0.000^**^
	Engineering	124 (21.27%)	117	7	
	Literature	118 (20.24%)	113	5	
	Medicine	142 (24.36%)	130	12	
	Business and Management	46 (7.89%)	43	3	
	Arts	55 (9.43%)	53	2	
Household registration	Urban household registration	299 (51.29%)	284	15	0.000^**^
	rural household registration	284 (48.71%)	264	20	
Family structure	single-parent family	155 (26.59%)	141	14	0.000^**^
	two-parent family	428 (73.41%)	407	21	

According to data analysis, college students’ depression has significant differences in gender, year, age, major, household registration and family structure. It is easy to find that female are more likely to suffer from depression than male. Freshmen and seniors students are more prone to depression, medicine major students having the highest depression rate, followed by engineering and natural science majors, then literature majors, arts as well as business and management majors has the lowest depression rate. College students with registered household in rural areas are more likely to suffer from depression than those in urban areas, and single parent families are more likely to suffer from depression than two-parent families.

### Descriptive statistics and correlation analysis

Pearson correlation analysis was conducted on self-consciousness, life meaning, self-efficacy, social support and depression (Table 2). Self-consciousness was negatively correlated with depressive emotions, also life meaning, self-efficacy, and social support were negatively correlated with depression. Self-consciousness, life meaning and self-efficacy are positively correlated. Research hypothesis 1 was validated. The correlation between these variables supported the subsequent hypothesis testing.

**Table 2:** Descriptive statistical results and correlation analysis between variables

Variables	M ± SD	Self-consciousness	Life meaning	Self-efficacy	Social support
Self-consciousness	41.59 ± 12.66	-			
Life meaning	48.86 ± 11.43	0.247^**^	-		
Self-efficacy	36.15 ± 7.15	0.417^**^	0.451^**^	-	
Social support	35.65 ± 7.68	0.173^**^	0.346**	0.326**	
Depression	43.84 ± 8.11	-0.354^**^	-0.231^**^	-0.309^**^	-0.307**

Note: **p< 0.01;*N* **= 583**

### Testing for mediating effects

**Fig. 2:**
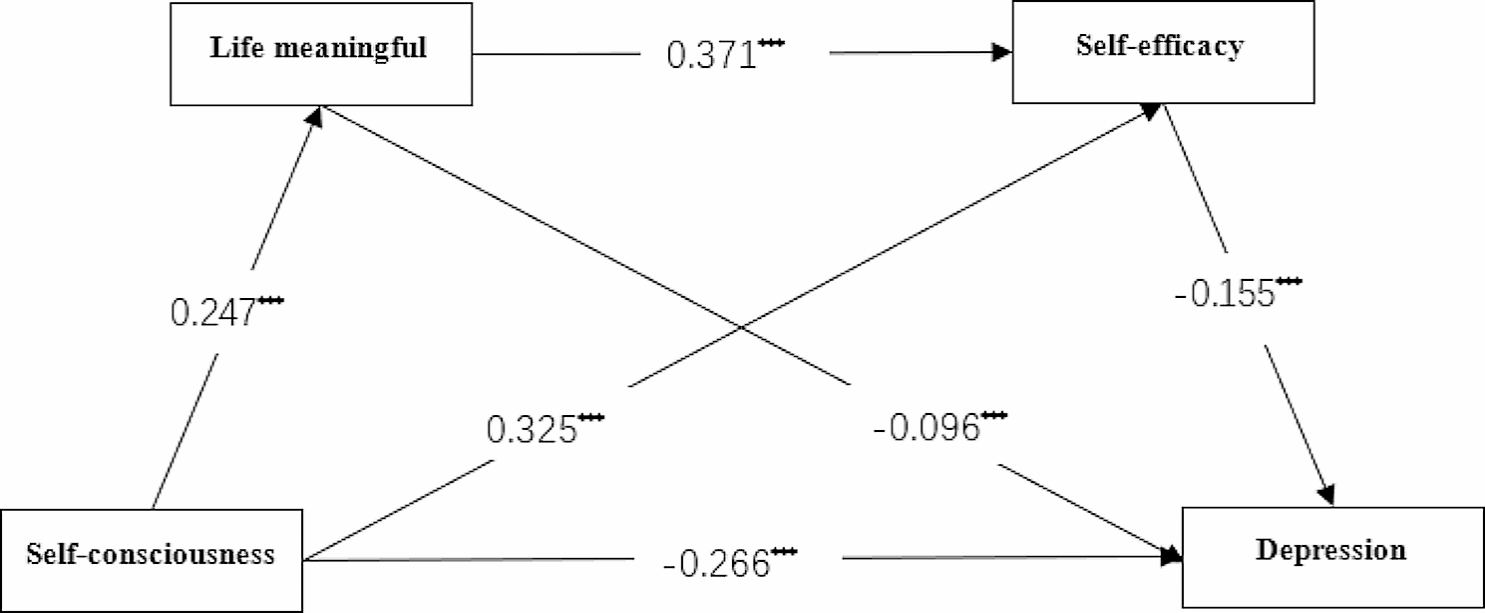
The mediation model. Path values are the path coefficients. All path coefficients were standardized. ***p<0.001

The bias corrected percentile bootstrap method was used to estimate the 95% confidence interval (CI) of the mediation effect and the moderation effect through 5000 sampling samples. The results have a positive significance when the CI does not include 0. First, the chain mediating effect of life meaning and self-efficacy between self-consciousness and depression was tested; then the moderating effect of social support on the path between self-efficacy and depression was tested. The chain-mediated effect results are shown in Fig. 2.

Based on the correlation analysis, the mechanism of self-consciousness influencing depression was examined using structural equation modeling, the constructed model is shown in Fig. 2. The model fitting results showed that the model was good for all indicators (${\chi }^{2}/df=4.98$, CFI = 0.95, RMSEA = 0.04, NNFI = 0.96, SRMR = 0.05). The results of mediating effect of life meaningful and self-efficacy are shown in Table 3. Self-consciousness had a significant negative effect on depression (*β* = -0.27, *SE* = 0.03, *t* = -6.36, *p* < 0.001, 95% CI = [-0.22, -0.12]), while self-consciousness had a significant positive effect on life meaningful (*β* = 0.25, *SE* = 0.04, *t* = 6.15, *p* < 0.001, 95% CI = [0.15, 0.29]) and self-efficacy (*β* = 0.33, *SE* = 0.03, *t* = 9.17, *p* < 0.001, 95% CI = [0.11, 0.24]), also life meaningful had a significant positive effect on self-efficacy (*β* = 0.37, *SE* = 0.02, *t* = 10.36, *p* < 0.001, 95% CI = [0.19, 0.28]). When the mediating variable life meaningful and self-efficacy were added, the direct effect of self-consciousness on depression was still significant (*β* = -0.25, *SE* = 0.02, *t* = -6.07, *p* < 0.001, 95% CI = [-0.19, -0.08]. Life meaningful (*β* = -0.10, *SE* = 0.03, *t* = -2.25, *p* < 0.05, 95% CI = [-0.13, -0.10]) as well as self-efficacy (*β* = -0.15, *SE* = 0.05, *t* = -3.40, *p* < 0.001, 95% CI = [-0.28, -0.07]) could also have a negative effect on depression. All three mediating effects were significant, as well as the total mediating effect, and it can be assumed that the mediating role of life meaningful and self-efficacy was established.

The mediating path was further tested and the results (see Table 4) showed that the total indirect effect value was − 0.088, 95% CI:[-0.130, -0.048], indicating that the model with life meaningful and self-efficacy as chain mediators was valid.

**Table 3:** Regression analysis of variable relationships in chain mediation models

The regression equation			Overall fitting index				Significance of regression coefficient			
Outcome variable	Predictors		*R*	*R* ^*2*^	*F*		*β*	LLCI	ULCI	*t*
Depression	Gender		0.38	0.14	39.76***		-0.06	-0.16	0.05	-1.28
	Age						-0.07	-0.14	0.03	-1.91
	Self-consciousness						-0.27	-0.22	-0.12	-6.36***
Life meaningful	Gender		0.35	0.12	31.89***		0.05	-0.10	0.08	1.65
	Age						0.06	-0.07	0.14	1.01
	Self-consciousness						0.25	0.15	0.29	6.15***
Self-efficacy	Gender		0.41	0.17	50.59***		0.04	-0.08	0.02	0.91
	Age						0.07	-0.11	0.13	1.21
	Life meaningful						0.37	0.19	0.28	10.36***
	Self-consciousness						0.33	0.11	0.24	9.17***
Depression	Gender		0.58	0.34	75.23***		-0.05	-0.08	0.03	0.91
	Age						-0.08	-0.15	0.07	-2.00
	Life-meaningful						-0.10	-0.13	-0.10	-2.25***
	Self-efficacy						-0.15	-0.28	-0.07	-3.40***
	Self-consciousness						-0.25	-0.19	-0.08	-6.07***

**Table 4:** Test of the chain mediation effect of life meaningful and self-efficacy on self-consciousness and depression

Effect	Paths	Std. Estimate	Boot std	Bootstrap 95% CI	Estimate
Direct effect	Self-consciousness→depression	-0.266	0.269	[-0.224, -0.118]	75.14%
Indirect effect 1	Self-consciousness→life meaning→depression	-0.024	0.108	[ -0.048, -0.005]	6.78%
Indirect effect 2	Self-consciousness→self-efficacy→depression	-0.050	0.017	[-0.085, -0.019]	14.12%
Indirect effect 3	Self-consciousness→life meaning→self-efficacy→depression	-0.014	0.006	[-0.027, -0.005]	3.95%
Total Indirect effect	/	-0.088	0.021	[-0.130, -0.048]	24.86%
Total effect	/	-0.354	0.025	[-0.276, -0.178]	100%

### Testing for moderated mediation

We examined the pathways between social support regulating self-efficacy and depression (Table 5). The results showed that self-efficacy had a significant predictive effect on depression (*β* = -0.17, *SE* = 0.04, *t* = -4.15, *p* < 0.001), social support had a significant predictive effect on depression (*β* = -0.20, *SE* = 0.03, *t* = -5.34, *p* < 0.001), and the interaction between social support and self-efficacy has a significant negative predictive effect on depression (*β* = -0.12, *SE* = 0.02, *t* = -3.13, *p* < 0.001). These findings suggest that social support as moderator was valid.

**Table 5:** The moderating role analysis of social support

Regression equation			Overall fitting index				Significance of regression coefficient			
Outcome variable	Predictors		R	R^2^	F		β	LLCI	ULCI	t
Self-efficacy	Gender		0.49	0.24	61.65^***^		-0.06	-0.16	0.05	-1.28
	Age						-0.07	-0.14	0.03	-1.91
	Self-consciousness						0.33	0.11	0.24	9.17***
Depression	Gender		0.25	0.05	22.13^***^		**-0.05**	**-0.15**	**0.06**	**-1.12**
	Age						**-0.06**	**-0.13**	**0.03**	**-1.28**
	Self-efficacy						**-0.17**	**-0.21**	**-0.07**	-4.15***
	Social support						**-0.20**	**-0.23**	**-0.08**	-5.34***
	Self efficacy×Social support						**-0.12**	**-0.10**	**-0.01**	-3.13***

**Fig. 3:**
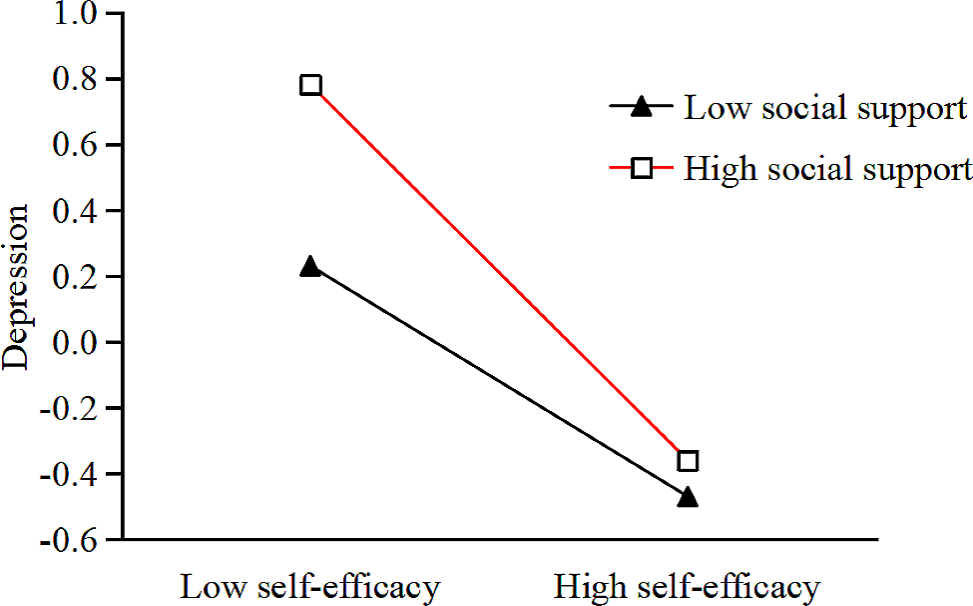
Depression as a function of self-efficacy and social support. The simple slope analysis indicated that social support moderated the relationship between self-efficacy and depression

To understand the essence of the moderating effect, we added one standard deviation to the social support score to form a high social support group and subtracted one standard deviation from the social support score to form a low social support group. The mediating effect of high social support was significant, with a total indirect effect value of 0.221 (*p* < 0.001). The mediating effect margin of the low social group was significant, with a total indirect effect value of 0.107 (*p* < 0.001). There was a significant difference in the mediating effect quantity between the two groups (*p* < 0.001). A simple slope analysis is shown in Fig. 3. For the low social group, self-efficacy had a significant negative predictive effect on depression (*b*_*simple*_ = -0.26, *t* = 8.9, *p* < 0.001, *R*^*2*^ = 0.11). For the high social support group, self-efficacy had a more substantial predictive effect on depression (*b*_*simple*_ = -0.45, *t* = 12.64, *p* < 0.001, *R*^*2*^ = 0.14).

## Discussion

Depression is the mental illness with the highest incidence worldwide. It is one of the five major causes of disability and disease [2]. Studying the influencing factors and mechanisms of adolescent depression is the foundation for scientific prevention and effective control and has significant theoretical and empirical value. The present study examined possible mechanisms through which self-consciousness influences depression. The findings highlighted several important points: (1) self-consciousness was negatively correlated with depression; (2) life meaningful and self-efficacy played sequential mediating roles in the relationship between self-consciousness and depression; (3) social support moderated the relationship between self-efficacy and depression.

### The mediating role of life meaning and self-efficacy

This study was done to investigate the role of self-efficacy as a mediator between self-consciousness and depression in college students, Consistent with previous research, self-consciousness was shown to have a substantial correlation with depression [39–41], Although some prior research did examine the role of mediators in self-consciousness and depression [42], there have hardly been any studies into the partial mediating role of self-efficacy and life meaningful in self-consciousness and depression. According to our findings, self-efficacy and life meaningful substantially partially mediates the relationship between self-consciousness and depression. The findings have supported the research hypotheses. These findings are consistent with prior research on the link between self-efficacy and depression [43, 44], the link between life meaningful and depression [45–47]. This study also explored how self-consciousness affects depression in college students. Research has found that self-consciousness can positively predict the sense of meaning in life. Improving college students’ self-consciousness means exploring and recognizing oneself, enhancing values and outlook on life [3]. The meaning of life includes the need for purpose, efficacy, fairness, and self-worth. Self-consciousness can enhance college students’ sense of meaning and self-efficacy by enhancing their sense of value, strengthening an understanding of the connection between oneself and the world, enhancing a sense of self-actualization identity, understanding the purpose and significance of life, and achieving life ideals and values with a positive attitude of dedication [2]. Students with solid life meaning can correctly understand their life’s value and meaning, often actively face difficulties and setbacks, and find ways to achieve goals, surpassing the consciousness of other classmates [48]. The awareness of mastering specific tasks through complex learning is robust, with stronger beliefs, information, and desires to achieve success, which promotes the formation of higher achievement motivation among young students and maintains an optimistic attitude [49].

### The moderating role of social support

Research revealed the moderating role of social support in the pathways of self-efficacy and depression. It is consistent with the previous studies. Self-efficacy and social support are important factors in the development and continuance of depression, both social support and self-efficacy significantly correlate with depression and self-efficacy partially mediates the relationship between social support and depression [50–52]. As a buffer resource for mental health, self-efficacy can mobilize an individual’s psychological resources, helping them adjust to a positive psychological state [39]. Social support can provide individuals with opportunities and resources to improve their self-efficacy [18]. While college students can naturally protect themselves and overcome difficulties when they can correctly view the problematic situations they encounter in realizing their dreams, negative emotions increase with the difficulty and intensity of setbacks [31]. Correcting overall confidence, such as general self-efficacy. When general self-efficacy is insufficient to correct negative cognition caused by environmental changes, at this time provide sufficient social support, college students will have a stronger sense of security and strength, reduce anxiety and unease, and can stimulate their desire to achieve success [33]. Laying a solid foundation for maintaining and improving self-efficacy among college students helps improve depressive symptoms [53].

### Implications

To reduce the level of depression among college students, schools and parents should attach importance to the cultivation and improvement of the sense of meaning in life among young students.

First, family should leverage the positive significance of family functions and factors that affect an individual’s sense of meaning in life. Interaction theory suggests that individual and environmental factors influence individual growth and development, and the external environment relies on seeking a sense of belonging and security in intimate relationships. Families can increase college students’ sense of belonging by establishing harmonious and intimate parent-child relationships [17]. For example, parents should respect their children’s choices and freedom, allowing them some time and space to independently do things they like. Chat with children more often, fully consider their opinions and ideas, and give them more opportunities to express and listen. When children express their own ideas and opinions, parents should listen carefully and provide responses and support as much as possible. Parents need to understand their children’s personalities and characteristics, be tolerant of their shortcomings, and actively praise their strengths.

Second, Universities should take appropriate measures and strategies to enhance and cultivate students’ sense of meaning in life. On the one hand, universities should offer courses related to the meaning of life and health. By increasing extracurricular volunteer service activities, organizing psychological capital themed groups, increasing individual success experiences, observing and imitating exemplary behaviors, and other activities, college students should be guided to understand the meaning of life from the learning process and specific events in life, and explore themselves in a targeted manner, enhance the general self-efficacy of college students and enhance their sense of meaning in life [2]. On the other hand, universities should provide various forms of social activities to promote interaction among students, including sports competitions, cultural and artistic festivals, classroom group learning, student union and volunteer services to provide students with a relaxed learning environment and good peer relationships.

Thirdly, attention should be paid to improving the self-efficacy of college students. For example, in response to non-student learning situations, teachers help students develop personalized phased learning goals, allowing them to see their progress in their studies and their progress in their studies [5]. Teachers can try to discovery the strengths of students, giving them ample opportunities to showcase themselves, and praising their bravery, kindness, optimism, allowing them to have more confidence and ultimately achieve success.

Finally, it is necessary to provide students with social support, such as providing spiritual encouragement and peer assistance in their studies, establishing a precise assistance system, providing timely material assistance for economically disadvantaged students, establishing special funds to slow down employment expenses, providing interview training, job recommendations, resume modifications, and other assistance to help students find employment. For students with interpersonal difficulties, art therapy [31], including psychological drama and painting therapy, can be offered to help them integrate into the group. The more social support an individual feels, the more channels they can access to relieve negative emotions, strengthen their happiness and satisfaction, and reduce depression.

### Limitations

There are some shortcomings in this study. First, this was a cross-sectional study exploring the impact mechanism of self-consciousness on depression in college students. Cross-sectional research has several advantages, including answering research questions and evaluating risk factors. As long as a test with high reliability and validity is selected, the results can support and explain complex models [54]. However, cross-sectional studies also have limitations, and future research should be designed in conjunction with longitudinal follow-up studies to explore the possible causal relationship between self-consciousness and depression in college students. Second, self-reported data were used by students. Although the common method bias in this study did not reach a significant level, future research should collect data from several channels (e.g., parents, teachers, and peers) to understand the relationships between variables.

### Conclusion

This study established a moderated mediation model to examine the influencing factors and mechanisms of depression in college students. The research analysis result showed that:

1. Self-consciousness can negatively predict the risk of depression in college students.
2. The meaning of life and self-efficacy mediate between self-consciousness and depression.
3. Social support plays a role in the mediating model of self-efficacy, self-consciousness, self-efficacy, and depression.

## Data Availability

Data will be available from the corresponding author on reasonable request.
